# Supplementary material for: Targeting HIV Env immunogens to B cell follicles in nonhuman primates through immune complex or protein nanoparticle formulations
Source: NPJ Vaccines. 2020 Aug 5;5:72. doi: 10.1038/s41541-020-00223-1 (PMC7406516; doi:10.1038/s41541-020-00223-1)
Supplement: Supplementary file 1 — Supplementary Information [file 41541_2020_223_MOESM1_ESM.pdf]

Supplementary Information:

Targeting HIV Env immunogens to B cell follicles in non-human primates through immune complex or protein nanoparticle formulations

Jacob T. Martin*[,1,2], Christopher A. Cottrell*[,2,3], Aleksandar Antanasijevic*[,2,3], Diane G. Carnathan[2,4,5], Benjamin J. Cossette[1,2], Chiamaka A. Enemuo[4,5], Etse H. Gebru[4,5], Yury Choe[4,5], Federico Viviano[4,5], Stephanie Fischinger[6,7], Talar Tokatlian[1,2], Kimberly M. Cirelli[2,8], George Ueda[9,10], Jeffrey Copps[3], Torben Schiffner[2,11], Sergey Menis[2,11], Galit Alter[6], William R. Schief[2,6,11], Shane Crotty[2,8,12], Neil P. King[9,10], David Baker[9,10,13], Guido Silvestri[2,4,5], Andrew B. Ward**[,2,3,11], and Darrell J. Irvine**[,1,2,6,13,14,15]

[1] Koch Institute for Integrative Cancer Research, Massachusetts Institute of Technology, Cambridge, MA 02139 USA.
[2] Center for HIV/AIDS Vaccine Development, The Scripps Research Institute, La Jolla, CA 92037 USA.
[3] Department of Integrative Structural and Computational Biology, The Scripps Research Institute, La Jolla, CA, 92037, USA.
[4] Yerkes National Primate Research Center, Emory University, Atlanta, GA 30322, USA.
[5] Emory Vaccine Center, Emory University School of Medicine, Atlanta, GA 30322, USA.
[6] Ragon Institute of Massachusetts General Hospital, Massachusetts Institute of Technology and Harvard University, Cambridge, MA 02139 USA.
[7] University of Duisburg-Essen, Essen 47057, Germany
[8] Center for Infectious Disease and Vaccine Research, La Jolla Institute for Immunology (LJI), La Jolla, CA 92037, USA.
[9] Department of Biochemistry, University of Washington, Seattle, WA 98195, USA.
[10] Institute for Protein Design, University of Washington, Seattle, WA 98195, USA.
[11] International AIDS Vaccine Initiative Neutralizing Antibody Center, The Scripps Research Institute, La Jolla, CA 92037 USA.
[12] Department of Medicine, Division of Infectious Diseases and Global Public Health, University of California, San Diego, La Jolla, CA 92037, USA.
[13] Howard Hughes Medical Institute, Chevy Chase, MD 20815 USA.
[14] Department of Biological Engineering, Massachusetts Institute of Technology, Cambridge, MA 02139 USA.
[15] Department of Materials Science and Engineering, Massachusetts Institute of Technology, Cambridge, MA 02139 USA.

*Equal contributions
**Correspondence to: djirvine@mit.edu, andrew@scripps.edu

Supplementary Table I. Cryo-EM data collection information

	BG505 SOSIP + RM19R Fab	BG505 SOSIP-T33_dn2 nanoparticle	
**Microscope**	Titan Krios	Talos Arctica	
**Voltage (kV)**	300	200	
**Detector**	Gatan K2	Gatan K2 Summit	
**Recording mode**	Counting	Counting	
**Magnification**	29,000 X	36,000 X	
**Movie micrograph pixel size**	1.03	1.15	
**Dose rate (e^{-}/Å^{2}/s)**	5.69	4.44	
**No. of frames per movie**	50	45	
**Frame exposure time (ms)**	250	250	
**Movie micrograph exposure time**	12.50	11.25	
**Total dose (e^{-}/Å^{2})**	67.0	50.0	
**Under focus range (μm)**	0.9 – 2.2	0.8 – 2.0	
**Number of movie micrographs**	1247	2748	
**Resolution (Å)**	3.71	4.60*	4.46[#]
**Number of particles**	191556	35521*	52939[#]
**EMDB**	EMD-21227	EMD-21231*	EMD-21230[#]

* Values/entries refer to the T33_dn2 nanoparticle core subparticle.
[#] Values/entries refer to the BG505 SOSIP antigen subparticle.

Supplementary Table II. Model refinement statistics

	BG505 SOSIP + RM19R	T33_dn2 Nanoparticle core	BG505 SOSIP Trimer
**PDB ID**	6VKN	6VL6	6VL5
**Residues**	2496	2640	1827
**Amino-acids**	2394	2640	1719
**Carbohydrates**	102	0	108
**RMSD Bonds**	0.018	0.019	0.018
**RMSD Angles**	1.690	1.457	1.712
**Ramachandran**			
**Favored (%)**	95.65	99.07	98.75
**Allowed (%)**	3.58	0.93	1.25
**Outliers (%)**	0.77	0.00	0.00
**Rotamer outliers (%)**	0.85	0.00	0.20
**Clash score**	1.05	0.28	0.61
**Molprobity score**	1.11	0.61	0.70
**EMRinger score**	3.33	0.21	1.55

Supplementary Figure 1:

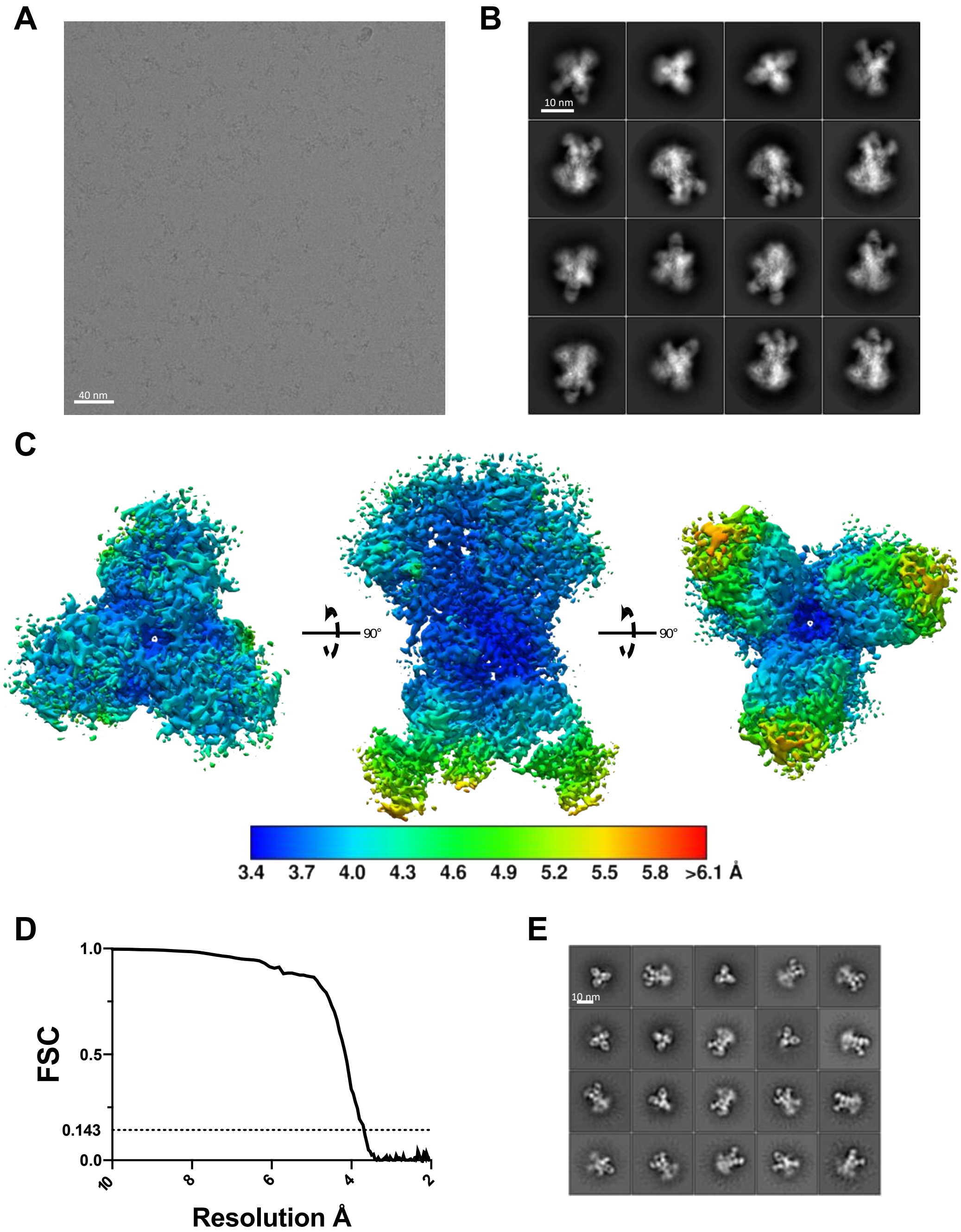

Supplementary Figure 1.

(A) Representative cryoEM micrograph of BG505 SOSIP.v5.2.N241.N289 trimer/RM19R Fab complex.

(B) 2D class averages of BG505 SOSIP.v5.2.N241.N289 trimer/RM19R Fab complex.

(C) Local resolution maps for BG505 SOSIP.v5.2.N241.N289 trimer/RM19R Fab complex.

(D) Gold-standard Fourier shell correlation (FSC) curves for BG505 SOSIP.v5.2.N241.N289 trimer/RM19R Fab complex showing global resolution calculated at FSC = 0.143.

(E) NS-EM 2D class averages of BG505 SOSIP.v5.2 following incubation with RM19R Fab at RT for >24 hrs.

Supplementary Figure 2:

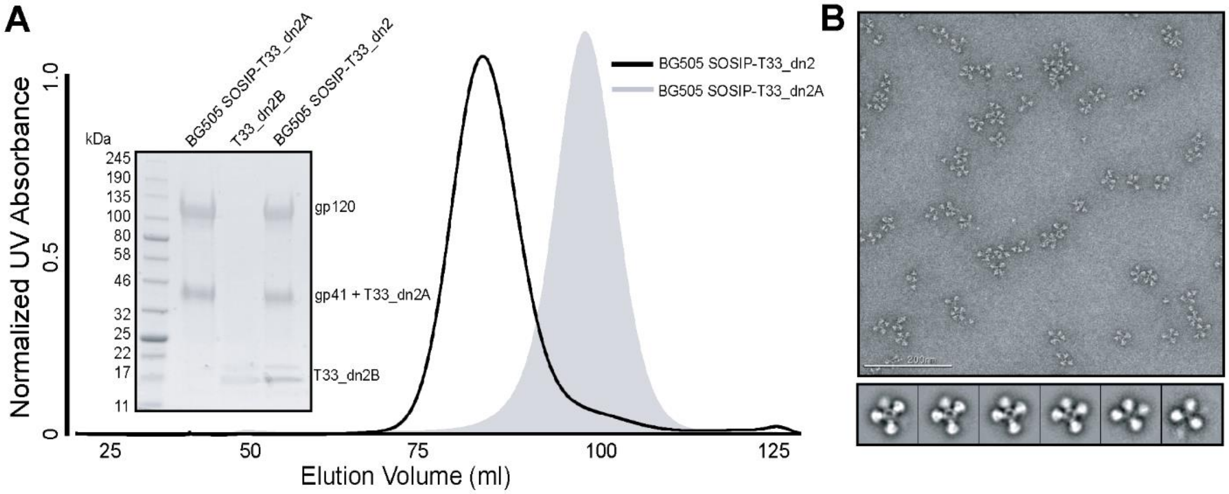

Supplementary Figure 2.

(A). Overlay of SEC chromatograms corresponding to BG505 SOSIP-T33_dn2A and assembled BG505 SOSIP-T33_dn2 nanoparticle and an SDS PAGE gel of the purified nanoparticle sample (B). Representative negative stain EM micrograph and 2D class averages of the assembled nanoparticle

Supplementary Figure 3:

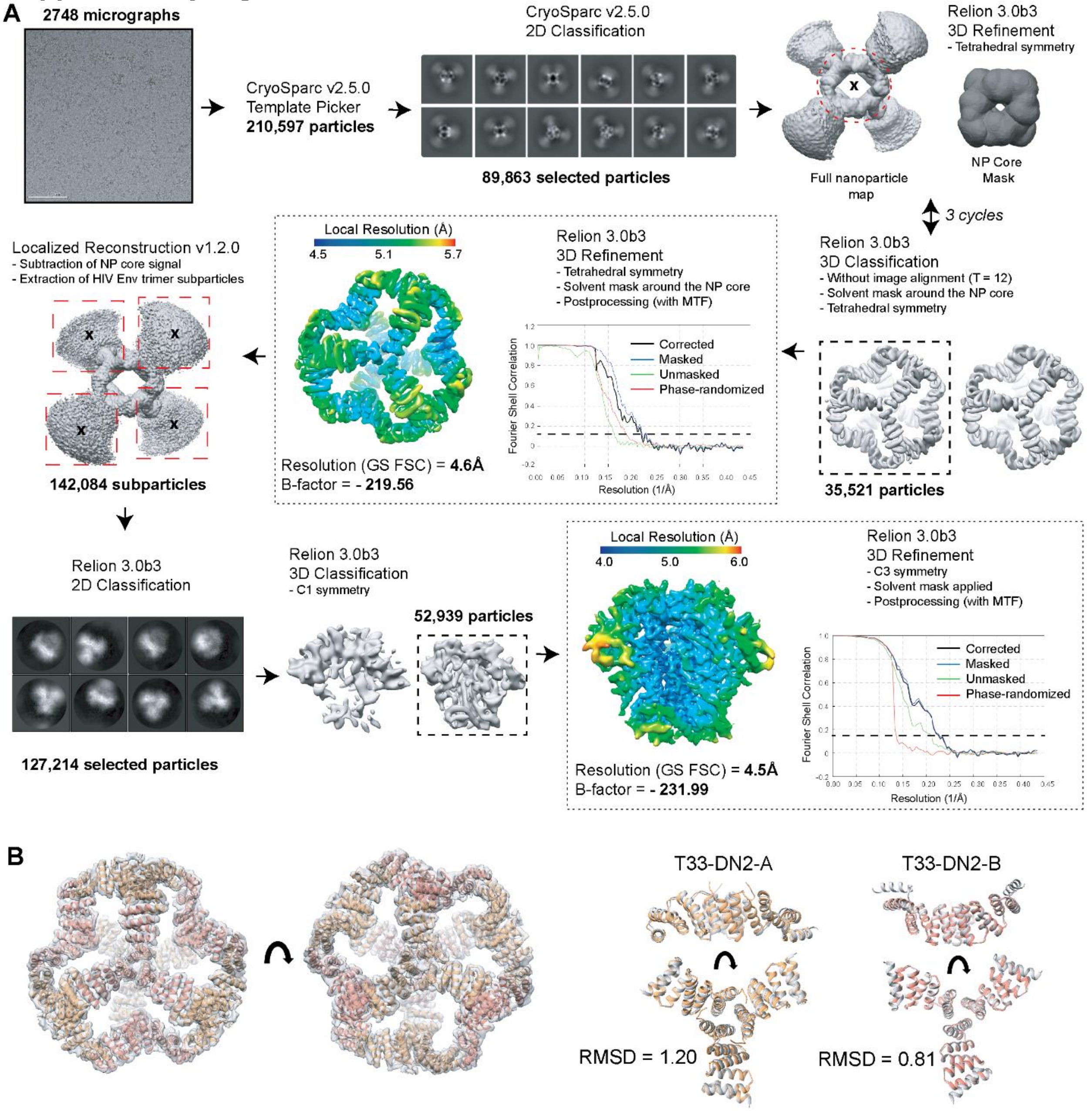

Supplementary Figure 3.

Cryo-EM data processing workflow for BG505 SOSIP-T33_dn2 nanoparticle dataset with relevant statistics (A). Fit of refined nanoparticle model to reconstructed density (left) and overlay of refined and Rosetta_design-predicted model of T33_dn2A and T33_dn2B (right) (B).

Supplementary Figure 4:

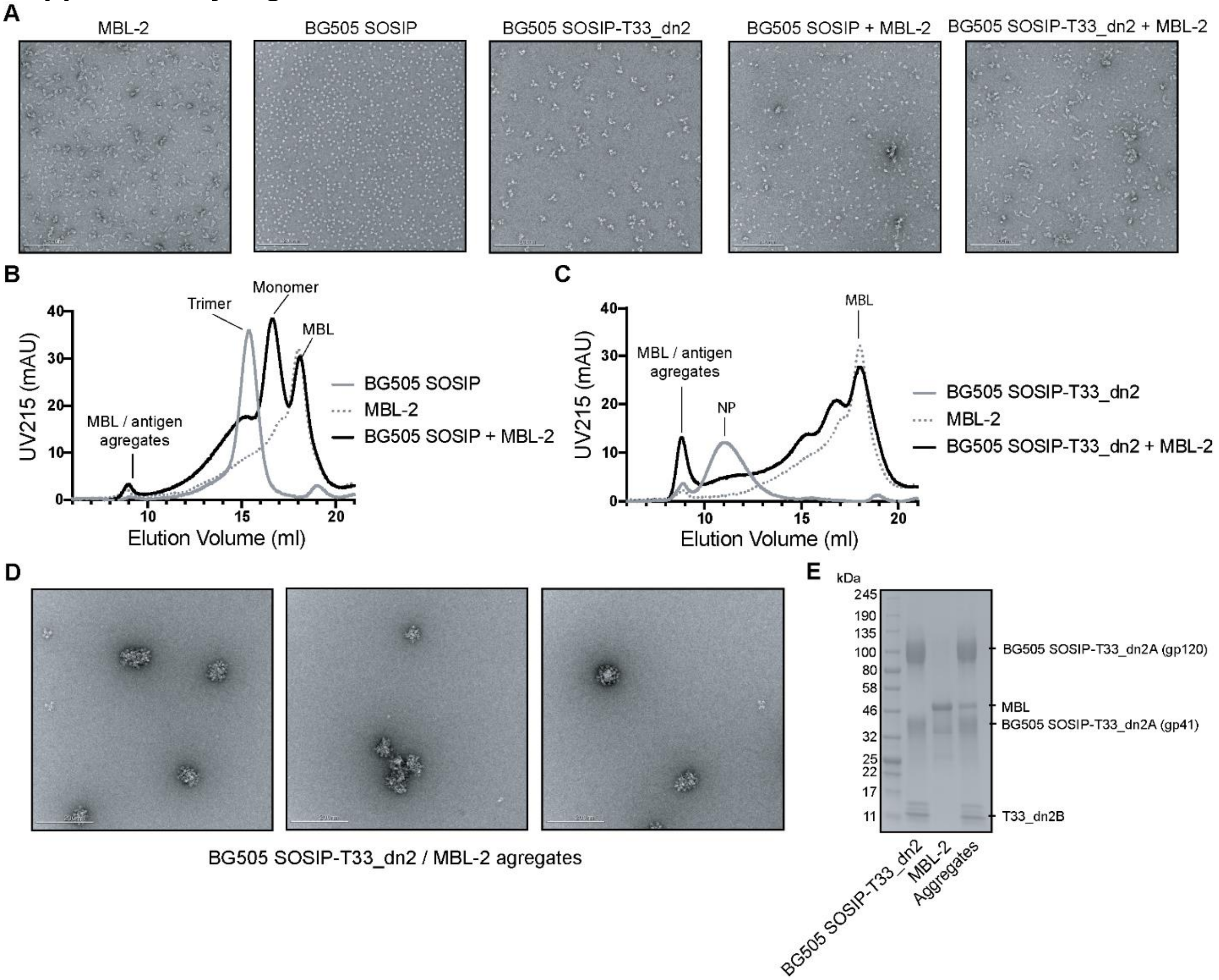

Supplementary Figure 4. MBL binding experiments.

(A) Human MBL-2 was incubated with free BG505 SOSIP and BG505 SOSIP-T33_dn2 nanoparticles for 4 hours at 37 °C. Negative-stain EM micrographs of the two samples are shown on the right. For comparison, EM micrographs of free MBL-2, BG505-SOSIP trimer and BG505 SOSIP-T33_dn2 nanoparticle are shown on the left. (B) SEC traces of free MBL-2, BG505 SOSIP and their combination following a 37 °C incubation. (C) SEC traces of free MBL-2, BG505 SOSIP-T33_dn2 nanoparticle and the combined sample following a 37 °C incubation. (D) SEC fractions corresponding to high-molecular weight aggregates in the MBL-2 + BG505 SOSIP-T33_dn2 sample were pooled, concentrated and imaged using NS-EM. Representative micrographs are shown. (E) SDS PAGE gel of free BG505 SOSIP-T33_dn2 nanoparticle, MBL-2 and the BG505 SOSIP-T33_dn2/MBL-2 aggregates purified by SEC.

Supplementary Video 1. 3D perspective and 360° rotation of cleared LNs from RM19R immune complex study.

Samples & data are the same as in **Fig. 3D**, displayed in three dimensions with rotation. Scale bars are 1 mm.

Supplementary Video 2. Close-up on follicles from cleared LNs from RM19R immune complex study, showing bowl-shaped morphology of follicular accumulation.

Samples are the same as in **Fig. 3D**, imaged at higher magnification (12.5x). Scale bars are 100 µm. Apparent changes in brightness during rotation are due to software interpolation in the z-plane (optical sections are spaced every 5 µm, resolution in x & y is 0.52 µm / pixel).

Supplementary Video 1. 3D perspective and 360° rotation of cleared LNs from trimer vs. nanoparticle comparison study.

Samples & data are the same as in **Fig. 5C**, with rotation. Scale bar is 5 mm.
