# Supplementary material for: Targeting HIV Env immunogens to B cell follicles in nonhuman primates through immune complex or protein nanoparticle formulations
Source: NPJ Vaccines. 2020 Aug 5;5:72. doi: 10.1038/s41541-020-00223-1 (PMC7406516; doi:10.1038/s41541-020-00223-1)
Supplement: Supplementary file 5 — Reporting Summary [file 41541_2020_223_MOESM5_ESM.pdf]

nature research

Corresponding author(s): Darrell J. Irvine
Andrew B. Ward

Last updated by author(s): Apr 30, 2020

Reporting Summary

Nature Research wishes to improve the reproducibility of the work that we publish. This form provides structure for consistency and transparency in reporting. For further information on Nature Research policies, see Authors & Referees and the Editorial Policy Checklist.

Statistics

For all statistical analyses, confirm that the following items are present in the figure legend, table legend, main text, or Methods section.

n/a	Confirmed	
☐	☒	The exact sample size (n) for each experimental group/condition, given as a discrete number and unit of measurement
☐	☒	A statement on whether measurements were taken from distinct samples or whether the same sample was measured repeatedly
☐	☒	The statistical test(s) used AND whether they are one- or two-sided<br>*Only common tests should be described solely by name; describe more complex techniques in the Methods section.*
☒	☐	A description of all covariates tested
☒	☐	A description of any assumptions or corrections, such as tests of normality and adjustment for multiple comparisons
☐	☒	A full description of the statistical parameters including central tendency (e.g. means) or other basic estimates (e.g. regression coefficient) AND variation (e.g. standard deviation) or associated estimates of uncertainty (e.g. confidence intervals)
☒	☐	For null hypothesis testing, the test statistic (e.g. F, t, r) with confidence intervals, effect sizes, degrees of freedom and P value noted<br>*Give P values as exact values whenever suitable.*
☒	☐	For Bayesian analysis, information on the choice of priors and Markov chain Monte Carlo settings
☒	☐	For hierarchical and complex designs, identification of the appropriate level for tests and full reporting of outcomes
☒	☐	Estimates of effect sizes (e.g. Cohen's d, Pearson's r), indicating how they were calculated

Our web collection on statistics for biologists contains articles on many of the points above.

Software and code

Policy information about availability of computer code

Data collection	Unicorn 7 software for chromatography, GE Healthcare Life Sciences Amersham Typhoon FLA9500 software for flatbed fluorescence scanner images, PerkinElmer Living Image software for IVIS, LaVision BioTec ImSpector Software for light sheet microscopy, Leica Application Suite X for confocal microscopy using a Leica sp8 microscope
Data analysis	GraphPad Prism 8.4.2 was used for plotting data and statistical analysis. FIJI package of ImageJ 1.52o for image processing. Microsoft Word and PowerPoint were used for manuscript preparation.

For manuscripts utilizing custom algorithms or software that are central to the research but not yet described in published literature, software must be made available to editors/reviewers. We strongly encourage code deposition in a community repository (e.g. GitHub). See the Nature Research guidelines for submitting code & software for further information.

Data

Policy information about availability of data

All manuscripts must include a data availability statement. This statement should provide the following information, where applicable:
- Accession codes, unique identifiers, or web links for publicly available datasets
- A list of figures that have associated raw data
- A description of any restrictions on data availability

Cryo-EM reconstructions have been deposited in the Electron Microscopy Data Bank (EMDB: EMD-21227, EMD-21230, and EMD-21231), and in the Protein Data Bank (PDB: 6VKN, 6VL5, and 6VL6).

Field-specific reporting

Please select the one below that is the best fit for your research. If you are not sure, read the appropriate sections before making your selection.

☒ Life sciences ☐ Behavioural & social sciences ☐ Ecological, evolutionary & environmental sciences

For a reference copy of the document with all sections, see nature.com/documents/nr-reporting-summary-flat.pdf

Life sciences study design

All studies must disclose on these points even when the disclosure is negative.

Sample size	Sample sizes of animals per group were chosen based on power analysis of expected results from prior related studies of antigen trafficking in large animals, and based on IACUC policies of reducing group sizes for animal research where possible.
Data exclusions	No data was excluded from analyses.
Replication	The macaque study has not been repeated due to costs associated with non-human primate research.
Randomization	Mice were randomly assigned to groups from a single shipment. Macaques were randomized by age and animal weights.
Blinding	Investigators were not blinded to sample groups in these studies.

Reporting for specific materials, systems and methods

We require information from authors about some types of materials, experimental systems and methods used in many studies. Here, indicate whether each material, system or method listed is relevant to your study. If you are not sure if a list item applies to your research, read the appropriate section before selecting a response.

Materials & experimental systems

n/a	Involved in the study	
☐	☒	Antibodies
☒	☐	Eukaryotic cell lines
☒	☐	Palaeontology
☐	☒	Animals and other organisms
☒	☐	Human research participants
☒	☐	Clinical data

Methods

n/a	Involved in the study	
☒	☐	ChIP-seq
☒	☐	Flow cytometry
☒	☐	MRI-based neuroimaging

Antibodies

Antibodies used	Macaque RM19R, and human PGT145 and VRC01 were produced in-house as described. Fluorescently-labeled Abs for staining commercially available, described in Methods as well as here: BV421 Rat Anti-Mouse CD35 Clone 8C12, BV421 mouse Anti-Human CD35 Clone E11, and Alexa Fluor® 488 Mouse anti-Ki-67 Clone B56 (BD Biosciences)
Validation	The commercially-available antibodies were validated by vendors (BD Biosciences). RM19R was the subject of this study. PGT145 and VRC01 were validated in the Alter lab.

Animals and other organisms

Policy information about studies involving animals; ARRIVE guidelines recommended for reporting animal research

Laboratory animals	Female Balb/C mice (JAX Stock No. 000651) 6 weeks of age, male and female Indian rhesus macaques varying ages between 2 years 6 months and 6 years 4 months of age
Wild animals	No wild animals were used in this study.
Field-collected samples	No field-collected samples were used in this study.
Ethics oversight	MIT IACUC, Emory University IACUC

Note that full information on the approval of the study protocol must also be provided in the manuscript.
